# Roles of Vascular Endothelial Growth Factor in Amyotrophic Lateral Sclerosis

**DOI:** 10.1155/2014/947513

**Published:** 2014-04-29

**Authors:** Ana Catarina Pronto-Laborinho, Susana Pinto, Mamede de Carvalho

**Affiliations:** ^1^Institute of Physiology, Faculty of Medicine, University of Lisbon, Avenida Professor Egas Moniz, 1649-028 Lisbon, Portugal; ^2^Instituto de Medicina Molecular (IMM), Translational Clinical Physiology Unit, Avenida Professor Egas Moniz, 1649-028 Lisbon, Portugal; ^3^Department of Neurosciences, Hospital Santa Maria, Centro Hospitalar Lisboa Norte, Avenida Professor Egas Moniz, 1649-028 Lisbon, Portugal

## Abstract

Amyotrophic lateral sclerosis (ALS) is a fatal devastating neurodegenerative disorder, involving progressive degeneration of motor neurons in spinal cord, brainstem, and motor cortex. Riluzole is the only drug approved in ALS but it only confers a modest improvement in survival. In spite of a high number of clinical trials no other drug has proved effectiveness. Recent studies support that vascular endothelial growth factor (VEGF), originally described as a key angiogenic factor, also plays a key role in the nervous system, including neurogenesis, neuronal survival, neuronal migration, and axon guidance. VEGF has been used in exploratory clinical studies with promising results in ALS and other neurological disorders. Although VEGF is a very promising compound, translating the basic science breakthroughs into clinical practice is the major challenge ahead. VEGF-B, presenting a single safety profile, protects motor neurons from degeneration in ALS animal models and, therefore, it will be particularly interesting to test its effects in ALS patients. In the present paper the authors make a brief description of the molecular properties of VEGF and its receptors and review its different features and therapeutic potential in the nervous system/neurodegenerative disease, particularly in ALS.

## 1. Introduction

Amyotrophic lateral sclerosis (ALS) is a devastating and fatal neurodegenerative disorder characterized by rapidly progressive degeneration of motor neurons in the spinal cord, brainstem, and motor cortex [1–4], leading to severe weakness and atrophy of voluntary muscles. However, it has heterogeneous clinical manifestations and variable progression rate [2–6]. ALS patients die mainly from respiratory failure, related to weakness of diaphragm and other respiratory muscles [7–9].

The median survival of ALS patients is 3–5 years after symptom onset [5, 10–12]. The worldwide incidence and prevalence are 2-3/100 000 and 4–7/100 000, respectively [13], with a male/female ratio of 1.5 : 1 [5, 10, 11, 14] approaching a 1 : 1 ratio in ALS patients with late symptom onset. Most patients have first symptoms between 55 and 65 years [5, 14].

Most ALS cases are sporadic (SALS) (90–95%) [6], but about 5–10% have a positive family history [6, 15]. Autosomal dominant inheritance is the typical pattern in familial ALS (FALS); however penetrance is quite variable. Approximately 20% of FALS are caused by a missense mutation in the gene encoding Cu/Zn super oxide dismutase (SOD1) [16]. More than 16 dysfunctional genes [17] have been described in FALS, including* alsin* (ALS2) [18, 19],* senataxin* (ALS4) [20],* vesicle associated membrane protein B* (VAPB, ALS8) [21],* angiogenin* [22, 23],* TAR DNA binding protein* (TARDBP or TDP-43) [24],* RNA-binding protein fused in sarcoma* (FUS) [25, 26],* optineurin* (OPTN) [27],* valosin-containing protein* (VCP) [28], and recently* C9ORF72* [12, 29, 30]. Genetics in ALS is far from simple; some gene mutations are of questionable value in the etiopathogenesis of the disease, as VAPB [31], and in some subjects different gene mutations should act synergistically in order to cause disease expression—oligogeniticity [32].

The pathophysiological mechanisms of motor neurons degeneration in ALS are complex, multifactorial, and incompletely known. Some of these mechanisms include abnormal RNA metabolism and transcriptional abnormalities, oxidative stress, excitotoxicity (induced by glutamate), protein aggregates, mitochondrial dysfunction, neuroinflammation, cytoskeletal derangements, deregulated growth factors, impaired axonal transport and apoptosis [6, 33], abnormal calcium metabolism, and activation of proteases and nucleases [2]. Riluzole is the only drug currently approved in ALS, acting by decreasing glutamate activity in the central nervous system. However, its impact on survival is modest [34]. Interestingly, trials with other antiglutamatergic drugs have been negative in humans [6, 35].

In 2001, Oosthuyse and coworkers associated vascular endothelial growth factor (VEGF) with the pathogenesis of ALS [36]. VEGF is a proangiogenic factor that confers neuroprotection by promoting neuron survival* in vivo* and* in vitro* [37–39], in particular increasing life expectancy of the ALS mice model [40–43]. In addition, it exerts protective effects on other cells, such as lung epithelia, bone marrow, and bone cells. Oosthuyse and colleagues studied VEGF in mice with a deletion of the hypoxia-response element (VEGF^*δ*/*δ*^). These mice developed adult-onset progressive motor neuron degeneration reminiscent of ALS [36] and showed reduced spinal cord and brain protein VEGF levels, suggesting that reduced hypoxia-mediated VEGF expression is associated with motor neuron degeneration.

In this review, we highlight the role of VEGF in ALS and its association with a potential therapeutic use.

## 2. Biology of VEGF

### 2.1. Members of the VEGF Family/Isoforms

VEGF is a homodimeric hypoxia-inducible glycoprotein, heparin-binding growth factor, which can induce angiogenesis* in vivo* [44], a potent mitogen, specific for vascular endothelial cells (ECs) [45, 46], but is also involved in some other processes such as inflammation and tumor progression [47]. In addition to hypoxia, other stimuli regulate the expression of VEGF gene, such as nitric oxide, estrogen, and a large variety of growth factors, like insulin-like growth factor (IGF-1), tumor necrosis factor alpha (TNF-*α*), epidermal growth factor (EGF), transforming growth factor beta (TGF*β*), interleukin- (IL-) 6, and IL1-*β* [45].

Presently, the VEGF family (see Table 1) comprises seven elements: VEGF-A, VEGF-B, VEGF-C, VEGF-D, PLGF (placental growth factor), VEGF-E (Orf-VEGF), and* Trimeresurus flavoviridis* svVEGF (snake venom VEGF). The ability of various isoforms of VEGF to bind heparin in laboratory assays depends on the presence or absence of two different heparin-binding domains [48].

#### 2.1.1. VEGF-A

VEGF-A is a dimeric glycoprotein with 33–42 kDa. Its gene expression is regulated by a variety of conditions such as hypoxia, growth factors, nitric oxide, p53 mutations, thyroid-stimulation hormone, and tumor promoters [49]. Higher levels of VEGF-A mRNA may be found in the adrenal gland, lung, kidney, and heart [50]. In humans, the gene encoding VEGF is located on the short arm of chromosome 6 (6p21.3) [51].

The VEGF-A gene has eight exons and seven introns [52, 53] and several homodimeric VEGF-A isoforms with biological activity have been identified: VEGF_121_, VEGF_121b_, VEGF_145_, VEGF_145b_, VEFG_148_, VEGF_165_ (predominant isoform), VEGF_165b_, VEGF_183_, VEGF_183b_, VEGF_189_, VEGF_189b_, and VEGF_206_ [54–57], respectively, composed of 121, 145, 148, 165, 183, 189, and 206 amino acid residues and resulting from alternative mRNA splicing [56–58].

#### 2.1.2. Placental Growth Factor (PLGF)

Placental growth factor (PLGF) is an angiogenic protein isolated from placenta, originally described in 1991 [59]. In humans, the PLGF gene is mapped on chromosome 14q24, and high levels of expression in placenta are found at all stages of gestation [49], as well as in the lungs, heart, skeletal muscle, and thyroid gland [49, 60, 61].

Four PLGF isoforms are produced through alternative splicing, with different sizes and binding properties: PLGF-1 (PLGF 131), PLGF-2 (PLGF 152), PLGF-3 (PLGF 203), and PLGF-4 (PLGF 224) [49, 62, 63]. PLGF-1 and PLGF-3 are nonheparin binding diffusible while PLGF-2 and PLGF-4 have heparin binding domains [63, 64]. These molecules play an important role in the regulation of vascular differentiation [65].

#### 2.1.3. VEGF-B

VEGF-B was discovered in 1996 [66]. Its gene is located on chromosome 11q13 [67] and comprises seven exons. Resulting from alternative splicing, two isoforms are expressed in human, VEGF-B_167_ and VEGF-B_186_ (of exon 6) [50], that bind to the tyrosine kinase VEGF receptor 1 (VEGFR-1). VEGF-B is a mitogenic factor for human ECs [68]. High levels can be observed in most tissues, in particular the neural tissues (retina, brain, and spinal cord) [69], myocardium, skeletal muscle, pancreas, prostate, and brown fat [68, 70–72].

Recently, VEGF-B has been extensively studied and considered as a strong cell survival factor for different types of cells, like neurons, vascular cells (pericytes, ECs and smooth muscle cells), and myocytes [69, 73, 74].

#### 2.1.4. VEGF-C and VEGF-D

Human VEGF-C gene is located on chromosome 4q34 [72] and the VEGF-D gene on chromosome Xp22.31 [75]. VEGF-C and VEGF-D isoforms result from proteolytic mechanisms, not from alternative splicing as VEGF-A and VEGF-B isoforms [70].

VEGF-C and VEGF-D have an important role in regulation of angiogenesis and lymphangiogenesis [72, 76–82] by promoting mitogenesis, migration, and survival of the ECs [49, 70, 83, 84]. Initially, premature forms of VEGF-C and VEGF-D are synthesized and subsequently activated when binding to receptors [70, 81, 85].

Human VEGF-C levels are higher in the placenta, ovaries, heart, small intestine, and thyroid gland while human VEGF-D levels are higher in the heart, lungs, skeletal muscle, colon, and small intestine [50].

#### 2.1.5. VEGF-E (Orf-VEGF)

VEGF-E consists of 120–140 amino acids [81]. It has a potent angiogenic activity, similar to VEGF-A [86], more potent in stimulating the proliferation of ECs than VEGF_165_, although having no heparin-binding basic domain [49].

#### 2.1.6. VEGF-F/Trimeresurus Flavoviridis svVEGF (T.f. svVEGF)

In recent studies, the VEGF family of proteins was also found in snake venom, including the svVEGF from* Bothrops insularis* [49, 87] and TfsvVEGF (Trimeresurus flavoviridis svVEGF) from pit vipers. Comprising 110–122 amino acid residues, svVEGF functions as dimmers [49]. TfsvVEGF strongly binds to VEGFR-1 but weakly to VEGFR-2, resulting in a weak angiogenic activity and a strong vascular permeability activity [81].

### 2.2. VEGF Receptors

VEGF binding sites have been identified on the surface of vascular ECs both* in vivo* and* in vitro* [52, 88]. Two distinct classes of VEGF receptors (VEGFR) exist—tyrosine kinases and nontyrosine kinase receptors [52, 55].

#### 2.2.1. VEGF Receptors Tyrosine Kinases

VEGFR family has three members (see Table 2): VEGFR-1 (fms-like tyrosine kinase-1 or Flt1), VEGFR-2 (KDR in humans, Flk1 in mice), and VEGFR-3 (fms-like-tyrosine kinase-4 or Flt-4). Both VEGFR-1 and VEGFR-2 contain seven immunoglobulin-like domains in the extracellular domain (VEGFR-3 contains only six), a single transmembrane region, and a consensus tyrosine kinase sequence interrupted by a kinase insert domain [50, 52, 89, 90].

VEGFR-1 and VEGFR-2 feature a central role in regulating angiogenesis, while VEGFR-3 stimulates lymphangiogenesis [52, 81, 91].

*VEGFR-1*. Human VEGFR-1 consists of 1338 amino acids [92] and is located on chromosome 13q12 [93]. VEGFR-1 binds not only to VEGF-A and VEGF-B but also to PLGF with high affinity. It is expressed in ECs, pericytes, placental trophoblasts, osteoblasts, renal mesangial cells, monocytes/macrophages, and some hematopoietic stem cells [52, 94]. It has a weak mitogenic action in ECs [70]. VEGFR-1 expression is upregulated during hypoxia by a hypoxia-inducible factor-1- (HIF-1-) dependent mechanism [95]. Interestingly, the soluble form of VEGFR-1 (soluble Flt-1) after undergoing alternative splicing inhibits VEGF activity [52, 96].

VEGFR-1 knockout mice die at embryonic day 8.5 to 9.0 stage (E8.5 to 9.0) from blood vessels disorganization and excessive growth, not from poor vascularization [70, 92]. This strongly suggests that VEGFR-1 offsets signals that induce angiogenesis by suppressing proangiogenic signals in the embryo (achieving a balance in physiological angiogenesis) [92].

In humans, abnormal high levels of soluble protein and sVEGFR-1 were found in serum in women with preeclampsia, accompanied by decreases in the circulating free PLGF and VEGF levels, possibly leading to EC dysfunction in the maternal and placental vessels [97, 98].

*VEGFR-2*. VEGFR-2 is located on chromosome 4q11→ q12 [99]. It binds to VEGF-A, VEGF-C, and VEGF-D and has strong tyrosine kinase activity, appearing to be the most important VEGF receptor, inducing mitogenesis, migration, and permeability of the ECs [50, 100, 101]. VEGF has higher binding affinity for VEGFR-2 than for VEGFR-1. Selective activation of VEGFR-1 and VEGFR-2 shows that the latter is the primary signal transmission of VEGF in ECs [52, 70].

VEGFR-2 activation induces production of platelet activating factor (PAF) by ECs, stimulating their mitosis and migration and increasing vascular permeability [50, 85]. It is also expressed in osteoblasts, cells of the pancreatic duct, neuronal cells, retinal progenitor cells, hematopoietic stem cells, and megakaryocytes. In nervous system, VEGFR-2 stimulates migration, proliferation, and survival of different neural cell types [90, 102, 103].

Inactivation of the VEGFR-2 gene in mouse resulted in the death of the embryo after E8.5 and E9.5, due to nondevelopment of embryonic vasculature, blood islands, and hematopoietic cells [104]. Therefore, VEGFR-2 signaling is essential for the development of the mouse embryo vessels [70, 92, 104–106].

*VEGFR-3*. VEGFR-3 binds to VEGF-C and VEGF-D [52, 81, 85, 91, 107]. Two human isoforms are described, resulting from alternative splicing and differing in their C-terminal [108].

Expression of VEGFR-3 begins at E8.5 in all embryonic mouse ECs. Afterwards, its expression is observed in developing venous and lymphatic vessels [70, 109, 110]. VEGFR-3 and their ligands play an important role in lymphangiogenesis [55, 111]. It is involved in mitogenesis, differentiation, and survival of lymphatic EC [107, 110, 112]. It is also expressed in osteoblasts, neuronal progenitor cells [113], and macrophages [111].

Hamada and coworkers showed that mice embryos with VEGFR-3 deficiency had abnormal vasculature and developed severe anemia and concluded that VEGFR-3 activity is critical in hematopoiesis and vasculoangiogenesis [114]. In another study, [115] it was observed that large vessel remodeling and maturation were abnormal in VEGFR-3 null mice embryos, causing abnormal fluid in the pericardial cavity and resulting in death after E9.5 by cardiovascular failure. The authors concluded that VEGFR-3 plays an essential role in the development of the cardiovascular system in an embryo before the emergence of lymphatic vessels [109, 111, 115].

#### 2.2.2. VEGF Receptors Nontyrosine Kinases

*Neuropilins: NRP-1 and NRP-2*. Neuropilin-1 (NRP-1) and neuropilin-2 (NRP-2) are nontyrosine kinase receptors. Neuropilins are single-spanning transmembrane glycoproteins of about 130–140 kDa, and have 45–50% sequence identity. NRP-1 and NRP-2 genes are located on chromosomes 10p12 and 2q34, respectively [116]. NRP-1 and NRP-2 bind to VEGF and PIGF. NRP-1 also binds to VEGF-B, whilst NRP-2 binds to VEGF-C [70, 116]. Both receptors bind to heparin-binding splice forms of VEGF: VEGF_165_ binds to NRP-1 and NRP-2; VEGF_145_ binds to NRP-2 [117–119]. Neuropilins potentiate VEGF signaling by acting as coreceptors for VEGF receptors [90, 120, 121].

These receptors play a fundamental role in the development of neuronal guidance, angiogenesis, and immunology and are also involved in cardiovascular system [116–118, 121, 122]. They belong to the class-3 semaphorin subfamily [117, 118, 122, 123], family of secreted molecules mediating repulsive signals in neuronal axon process guidance [116]. In a chimeric mice study, overexpression of NRP-1 caused excessive blood vessel formation and heart malformation [116, 124].

In a recent study, knockout NRP-1 mutant mice died at embryo stage E13 from impaired neural vascularization, transposition of large vessels, and weak vascular network development in the yolk sac. Therefore, the authors concluded that NRP-1 is both effective in embryonic vessels formation and in nerve fiber guidance [125]. A study in zebrafish model underscored that NRP-1 is required for the VEGF-dependent angiogenesis [46, 126]. A study with NRP-2 knockout mice demonstrated severe abnormalities in the development of capillaries and small lymphatics [127]. In NRP-1 and NRP-2 double knockout mice, the embryonic mice died at E8.5 with avascular yolk sacs [128].

### 2.3. Regulation of VEGF Expression

VEGF expression is stimulated by several factors, such as oncogenes, thyroid-stimulating hormone, estrogen, nitric oxide (NO), hypoxia, and growth factors. VEGF gene contains various potential binding sites for transcriptional regulators, like activator protein-1 (AP-1), activator protein-2 (AP-2), HIF-1, specific protein (SP-1), Egr-1, signal transducer, and activator of transcription-3 (Stat-3) [55, 129].

We will shortly mention the key factors and conditions related to regulation of VEGF expression.

#### 2.3.1. Oxygen Tension

Erythropoietin (EPO) is a glycoprotein hormone/growth factor. Its gene is localized on chromosome 7 (7pter-q22) [130]. EPO-producing cells, localized in the liver and kidneys, have the ability to sense oxygen concentration and respond to systemic hypoxia by increasing EPO gene transcription [95, 131, 132]. EPO controls erythropoiesis and, thereby, oxygen transportation by circulating erythrocytes.

Reduced local oxygen tension induces angiogenesis [133], by regulating the expression of a variety of genes. In particular, VEGF mRNA expression is stimulated [46, 134]. Moreover, VEGF production is upregulated by hipoglicemia and acidosis [129, 131, 135]. Hypoxic transcriptional regulation of VEGF-A is mediated by HIF-1, a heterodimeric protein transcription factor [136]. Indeed, HIF-1 is also activated by other environmental stresses as low pH, cytokines, and several growth factors [137].

HIF-1 has two subunits, HIF-1 alfa (120-kDa) and HIF-1 beta (91–94-kDa), both being basic-helix-loop-helix Per-aryl hydrocarbon receptor nuclear translocator-Sim (PAS) family of transcription factors [138–141]. Under normoxic conditions, HIF-1 alfa (HIF-1*α*) is degraded by ubiquitin pathway (proteasome degradation-26S proteosome) [142, 143]. HIF-1*α*, hydroxylated by prolyl hydroxylases proteins (PHDs), interacts with the von Hippel-Lindau (pVHL) protein, a component of the E3 ubiquitin ligase complex, marking HIF-1*α* for degradation [144–146]. However, under hypoxic conditions, PHDs are inactive, HIF-1*α* increases dramatically, and the fraction that is ubiquitinated decreases [143, 147]. HIF-1*α* translocates into the nucleus and associates with HIF beta (HIF-*β*) and the coactivators p300/CBP to induce gene expression by binding to the conserved [A/G] CGTG hypoxia-responsive element (HRE) [148]. Hypoxia promotes the induction of transcription and the stabilization of VEGF mRNA through proteins that bind to sequences located in the region UTR (3′unstranslated region) of the VEGF mRNA [149].

#### 2.3.2. Cytokines and Growth Factors

VEGF expression in different cells is upregulated by cytokines (IL-1*α*, IL-1*β*, IL-6, and TNF-*α*), prostaglandins (E1 and E2), and numerous growth factors (transforming growth factor *α*, transforming growth factor *β*, epidermal growth factor, fibroblast growth factor 4, keratinocytes growth factor, insulin-like growth factor-1, platelet-derived growth factor BB, and basic fibroblast growth factor) [46, 136, 150–159]. The upregulated VEGF mRNA expression suggests that paracrine or autocrine release of such factors acts synergistically with local hypoxia to increase VEGF release [46, 52, 65, 149], with influence on angiogenesis/permeability in inflammatory disorders [46, 52, 149, 150].

#### 2.3.3. Tumor Suppressor Genes and Oncogenes

The involvement of altered oncogenes and suppressor genes is crucial. The uncontrolled typical tumoral growth results from activation of oncogenes and loss of genes/tumor suppressor proteins at different sites in cell signaling pathways [160]. Oncogenic mutations or amplification of Ras results in VEGF upregulation. Some studies indicate that Ras-dependent VEGF expression is necessary, although insufficient, for progressive tumor growth* in vivo* [52, 161].

The overexpression of the VEGF gene can occur due to a mechanism of inactivation of the tumor suppressor gene. The von Hippel Lindau (VHL) tumor suppressor gene was implicated in the regulation of VEGF gene expression [162]. VHL protein provides a negative regulation of a group of hypoxia-inducible genes, such as VEGF, platelet-derived growth factor B chain, and the glucose transporters GLUT-1 genes [163]. In presence of a mutant VHL, mRNAs for such genes were produced both under normoxic and hypoxic conditions [65].

## 3. VEGF in the Nervous System

VEGF has an important role in vascular growth regulation and development. Moreover, VEGF produces direct effects in a variety of neuronal cells, including neuritic outgrowth, axon guidance, neuronal survival, and neuronal migration. Recent studies have shown that in adverse conditions for neuronal cells, such as oxidative stress, hypoxia, or glucose deprivation, VEGF acts as a mediator of multiple mechanisms inhibiting cell death and stimulating neurogenesis [36–38, 90, 103, 164–168].

### 3.1. VEGF in Neurogenesis

Production of progenitor cells and neurons occurs during development and in adult age. The two fundamental regions contributing to neurogenesis in the adult mammalian brain are the subventricular zone (SVZ) of the lateral ventricles and the subgranular zone (SGZ) in dentate gyrus of the hippocampus [169]. In both regions, neurogenesis occurs in the vicinity of blood vessels by the proliferation of neural stem cells (NSCs) in small clusters [90, 167, 170].

In adult brain, neurogenesis is a dynamic process regulated by several stimuli, in particular growth factors (epidermal growth factor, EPO, brain-derived neurotrophic factor, stem cell factor, and VEGF), glucocorticoids, sex hormones, and excitatory neurotransmitters [164]. It can also occur in response to critical situations such as cerebral ischemia, mechanical trauma, and seizures [164, 166, 168, 169, 171]. In addition, these injuries drive migration of new neurons into affected regions to promote tissue repair [171].

VEGF has an important role in adult neurogenesis [90, 172], participating in the crosstalk between ECs and NSCs within vascular niches. This process can be highlighted by the presence of cells labeled with bromodeoxyuridine (BrdU) and doublecortin (Dcx), which are markers of proliferation and neuronal lineage, respectively [171]. In animal studies, intracerebroventricular administration of VEGF stimulates neurogenesis. Additionally, other studies have stressed that VEGF favors survival of newly formed neurons [103, 164, 166]. The role of VEGF in neurogenesis is rather complex. Testosterone-induced neurogenesis is preceded by an upregulation of VEGF and VEGFR-2. Brain-derived neurotrophic factor (BDNF), secreted by ECs, acts through VEGF increase subsequently stimulating neural cell proliferation [173]. Granulocyte colony-stimulation factor (G-CSF) stimulates neurogenesis by increasing the release of VEGF from NSCs and the expression of VEGFR-2 in those cells. VEGFR-2 tyrosine kinase inhibitor blocks the neurogenesis stimulated by G-CSF [172, 174].

Antidepressant drugs stimulate neuron proliferation in part due to the upregulation of VEGF and VEGFR-2 signaling in the hippocampus [175]. This positive effect is lost by previous intraventricular infusion of a VEGFR-2 inhibitor. In behavioral models, VEGF infusion into the brain mimics the positive effect of antidepressants drugs on neurogenesis [167, 175–177]. Hippocampus VEGF expression is increased in animals exposed to enriched environment conditions and on learning protocols. The hippocampal overexpression of VEGF via viral gene transfer led to increased neurogenesis and improved cognition. In a study, the use of a dominant-negative mutant KDR/VEGFR-2 (mKDR) resulted in negative effects on neurogenesis and learning process, but not inhibiting the proliferation of ECs [172, 178].

VEGF-B participates in the regulation of neurogenesis in adult brain. Intracerebroventricular VEGF-B administration leads to BrdU incorporation in neuronal lineage cells* in vivo* and* in vitro.* Moreover, VEGF-B knockout mice showed reduced neurogenesis, coherent with its neurogenesis-promoting activity [165, 179]. VEGF-C promotes neurogenesis of neural progenitor cells expressing the VEGFR-3 [113]. Overexpression of VEGF-C stimulates VEGFR-3 expressing NSCs as well as neurogenesis in the SVZ, but not affecting angiogenesis. Reciprocally, SVZ neurogenesis is diminished by conditional deletion of VEGFR-3 in neural cells and by blockage of VEGFR-3 signaling with antibodies [180].

### 3.2. VEGF in Neuroprotection and Neuroregeneration

Experimental studies* in vivo* and* in vitro* demonstrated the neuroprotective, neurotrophic, and angiogenic properties of VEGF [36, 37] through its action on various neuronal cell types [42, 43, 90, 166]. The effects are observed in hippocampus, as well as in dopaminergic, cerebellar, and retinal neurons, and also in the peripheral nervous system. VEGF protects these cells against death induced by a wide variety of different stress conditions like hypoxia and excitotoxicity stimuli [37, 42, 90, 171, 181, 182]. In hippocampus, protection against excitoxicity occurs via two pathways: phosphatidylinositol 3-kinase (PI3K)/Akt (PI3K-K/AKT) and mitogen-activated protein kinase/extracellular signal-regulated kinase (MEK)-extracellular signal regulated kinase (ERK) (MEK/ERK) [183]. VEGFR-2 is predominantly responsible for mediating this neuroprotective effect, although VEGFR-1 can also have some of these effects [90].

Some studies demonstrated that VEGF exerts its neuroprotective action directly by inhibiting programmed cell death (apoptosis). VEGF also exerts neuroprotective effects indirectly through its action on multiple processes such as angiogenesis, increased blood brain barrier permeability to glucose and activation of antioxidants [90, 181, 184].

#### 3.2.1. Direct Neuroprotective Functions of VEGF

Several studies support the role of VEGF in increasing neuron survival when subjected to hypoxia, by inhibition of Caspase 3 activation via Caspase 9, thereby reducing apoptosis [103, 164, 166, 184–186]. The increased expression of VEGFR-2 in neurons, when subjected to hypoxia and glucose deprivation, indicates that this receptor is especially involved in the neuroprotective effect of VEGF [184, 186, 187].

#### 3.2.2. Indirect Neuroprotective Functions of VEGF

VEGF also has an indirect neuroprotective function that affects neuronal survival in critical conditions. It promotes heme oxygenases antioxidative activity [188–190], increases brain glucose supply by GLUT-1 transport pathway, dilates cerebral arterioles, and induces endothelial fenestrations [191]. Moreover, angiogenesis exerts neuroprotective effects by increasing cerebral blood flow [37, 184].

VEGF has neuroregenerative properties. Bearden and colleagues used a model of axotomy to show regeneration mediated by VEGFR-2 activation. In addition, VEGF sequestration by sVEGFR-1 impairs the regeneration of axotomized nerves [192, 193]. VEGF promotes increased growth cone area of sympathetic neurons via VEGFR-2 and NRP-1 mediation [193]. Other studies support VEGFR-1 signaling induction of chemotaxis and microglial proliferation [194]. VEGF also acts on glial cells in the adult nervous system, promoting neuron survival [39, 195, 196].

The various roles of VEGF proteins in the nervous system of the adult brain are briefly summarized in Tables 3 and 4.

## 4. Role of VEGF in ALS

### 4.1. Animal Models

#### 4.1.1. Rats and Rodents Models

Strong evidence of the role of VEGF in the nervous system comes from studies in ALS. In the past decade, VEGF has been implicated in motor neuron degeneration in mice. In 2001, Oosthuyse and colleagues found that transgenic mice with homozygous deletion of the hypoxia-response element in the VEGF promoter (VEGF *δ*/*δ* mice) gene showed reduced VEGF levels by about 25–40% in the neural tissue. The affected mice developed limb weakness and neurogenic muscular atrophy by five weeks of age, with electromyographic signs of muscle denervation and reinnervation associated with loss of motor neurons in the spinal cord. Approximately 60% of the mice died before or at birth due to severe lung vascular alterations [36, 42, 181, 197, 198]. A follow-up study [40] used mice that resulted from crossing SOD1^G93A^ and VEGF^*δ*/*δ*^ models [199]. This mice model showed an earlier onset of muscle weakness and shorter survival [40]. Wang et al. crossed SOD1^G93A^ mice with mice overexpressing VEGF-A in neurons. These double transgenic mice had a significant delay in motor neuron loss and in the onset of weakness, resulting in an increased survival compared to the single transgenic mice [3].

Interestingly, decreased VEGF expression in the spinal cord of SOD1^G93A^ mice has been reported.* In vitro* models suggest that it derives from reduced stability of mRNA VEGF. Indeed, Lu and colleagues identified that mutant SOD1 leads to downregulation and destabilization of mRNA VEGF [200]. It was shown that in mutant SOD1 mice but not in wild-type mice, ribonucleoprotein complex with adenine and uridine-rich stability elements (ARE) is present in the 3′unstranlated region (UTR) of mRNA VEGF, resulting in lower VEGF expression levels [200–202]. Contrary to the above reports, Van Den Bosch et al. described no significant difference in the levels of VEGF-A in the spinal cord of SOD1^G93A^ mice when compared to wild-type controls [203] and Murakami et al. reported a higher VEGF expression level in the spinal cord of SOD1^G93A^ mouse [204]. It seems that VEGF expression in SOD1^G93A^ motor neurons is minimally upregulated by hypoxia [90, 204], which reinforces the role of VEGF expression in the pathogenesis of motor neuron degeneration [204]. Exposition of mutant SOD1^G93A^ mice to prolonged periods of hypoxia did not affect their life span after the upregulation of VEGF in the spinal cord [203].

Resulting from the VEGF role in perfusion of the brain and spinal cord [36, 172, 203, 205] a reduction of neuronal perfusion in the symptomatic VEGF^*δ*/*δ*^ mice was described [36]. Moreover, decreased spinal cord blood flow has been observed in the presymptomatic mutant SOD1 mice [8, 36, 206]. However, it still remains unclear whether the reduced neural perfusion is present before the onset of motor neuron degeneration or is just a consequence of neuronal loss.

As most trophic effects on motor neurons are mediated by VEGFR-2 and NRP-1 [42, 207, 208], several authors suggest that coexpression of VEGF and VEGFR-2 may lead to supplementary positive effects on these cells [36, 209, 210]. When the transgenic Thy:VEGFR-2 mice, which leads to an overexpressing VERGFR-2 under control of Thy1.2 promoter that triggers its expression in postnatal neurons, were intercrossed with SOD1^G93A^, the resulting double transgenic mice, Thy-VEGFR-2 X SOD1^G93A^, demonstrated that VEGFR-2 overexpression in motor neurons retards degeneration of spinal motor neurons in SOD1^G93A^ mice [90, 211]. A recent study showed that the administration of recombinant VEGF_165b_ exerts neuroprotection against multiple insults. The neuroprotection is dependent on the activation of the VEGFR-2 and MEK1/2, and the inhibition of caspase 3 induction [212].

#### 4.1.2. Zebrafish Models

In zebrafish embryos, the overexpression of mutant SOD1 (mSOD1) protein revealed the induction of a dose-dependent motor axonopathy. Lowering VEGF induced a more severe phenotype, whereas upregulating VEGF rescued mSOD1 axonopathy [213].

More recently, Da Costa created a new model of zebrafish, the T70I mutant (mutant zebrafish line). This mSOD1 protein zebrafish replicates several of the features of human ALS, such as early neuromuscular junction abnormalities, adult-onset motor neuron disease phenotype, and susceptibility to oxidative stress. This model can be a useful* in vivo* tool for therapeutic screening [214].

#### 4.1.3. Culture Models

It was observed in a study using motor neuron models, in cultures containing mutant SOD1^G93A^, through administration of adenovirus vector, that VEGF increased neurons survival [215]. Tovar-y-Romo and Tapia showed the importance of the PI3K/AKT survival pathway role, as well as the inhibition of p38 MAPK (p38 mitogen activated protein kinase)—stress-activated protein kinase, in the protective mechanism exerted by VEGF against excitotoxic spinal motor neuron death* in vivo*, thus suggesting that VEGFR-2, the p38 MAPK, and PI3K pathway constitute appealing therapeutic targets for ALS [208].

Increase in motor neuron survival by VEGF has been reported even in adverse conditions such as hypoxia, hypoglycemia, and deprivation of serum. However, it seems that VEGF does not protect neurons from excitotoxicity [203].

### 4.2. VEGF-B in ALS

VEGF-B, recently identified by Grimmond et al. (1996) [66] and Olofsson et al. (1999) [72], appears to provide a potent survival and protective factor for neurons, namely, cortical, retinal, and spinal cord motor neurons [165, 216, 217]. Moreover it is a survival factor for endothelial cells, pericytes, and smooth muscle cells [201, 218, 219]. Administration of small quantities of VEGF-B causes neuroprotection without causing adverse effects or vascular blood-brain barrier leakiness [220]. In contrast, VEGF-A administration is related to angiogenesis and edema [220, 221]. In VEGF-B in knockout mice, Sun and colleagues described a 40% increase of lesion size after cerebral ischemia. It was also found that VEGF-B protected cultured cortical neurons from hypoxic brain injury [165]. More recently Li and coworkers [201, 217, 219] showed that VEGF-B acts as a potent survival factor by suppressing the expression of BH3-only protein and other apoptotic cell death-related genes [201, 217, 219].

When crossing SOD1^G93A^ with VEGFB^−/−^ mice two lines SOD1^G93A^ VEGF-B^+/−^ and VEGF-B SOD1^G93A^ VEGF-B^−/−^ are obtained. Double transgenic mice SOD1^G93A^ VEGF-B^−/−^ developed the most severe form of motor neuron disease [211, 220, 222]. Further treatment with VEGF-B has changed clinical phenotype to a milder form, as observed for VEGF-A [211]. Since VEGF-B shows weaker angiogenic and permeability properties than VEGF-A, it has an excellent safety profile.

### 4.3. VEGF in Human ALS

#### 4.3.1. Genetic Studies

Several clinical studies point to the involvement of VEGF in ALS. However, clinical reports failed to identify any specific VEGF mutation [40, 223–225]. A study based on Swedish, English, Belgian, Russian, and American patients displayed an association between 2 haplotypes determined by 3 single-nucleotide polymorphisms (SNPS) −2.578 C/A, −1.154 L/C, and −634 G/C—in the VEGF upstream promoter/leader sequence of the VEGF gene with an increased risk of ALS [40, 226–228]. Other studies reported no association between these polymorphisms and SALS in various populations, as in Chinese [229], Italian [230], Dutch [231], German [232], British [224], and North American [233]. A meta-analysis was performed in order to clarify those different results: three polymorphisms were studied, involving 7000 patients from various countries in Europe and America. This study did not confirm the original conclusion that VEGF haplotypes increase the risk of ALS in humans but confirmed a significant association of the VEGF-2578 AA genotype and ALS susceptibility in males [226].

#### 4.3.2. Plasma, Serum, and Cerebral Spinal Fluid (CSF)

There are conflicting reports of VEGF levels in CSF, serum, and plasma in humans with ALS. Normally, VEGF levels are much higher in serum than in plasma and even lower in CSF. Measurements in serum are potentially contaminated by release of VEGF from platelets [234]. Serum levels of VEGF in ALS have been reported as unaltered [235] or higher than normal [236, 237]. Lambrechts et al. [226] found that plasma VEGF levels were around 50% lower in ALS patients than in controls, in particular in those carrying at-risk* VEGF* genotypes. However, in other studies plasma levels of VEGF were within normal limits in ALS patients [237–240] without significant change in patients with hypoxemia [237, 239, 240]. Indeed, Moreau and coworkers showed that HIF-1 was not activated by hypoxia in monocytes from ALS patients [241].

Devos et al. [238] studied CSF VEGF levels in 24 patients with ALS. They reported lower CSF levels in the early phase of the disease when compared with other disease controls, but similar to levels recorded in healthy subjects. In contrast, Gupta et al. [242] found that VEGF levels were increased in CSF and serum in ALS in general and further increased with hypoxia. In long duration ALS, Ilzecka [243] found that the VEGF level in CSF was increased. The VEGF level in CSF is not clearly associated with hypoxia in ALS, as a lack of upregulation of CSF VEGF during hypoxemia has been reported, when compared with hypoxemic controls [244].

Carilho et al. found no correlation between VEGF plasma levels and forced vital capacity, PaO_2_ or PaCO_2_, meaning that hypoxia was not an effective stimulant of VEGF production. However, it was reported that ALS patients in respiratory distress showed a marked increase in VEGF level following noninvasive ventilation onset. This suggests that correction of hypoxia leads to higher activation of the hypoxia element response in the VEGF promoter by HIF-1. In addition, the same authors found that VEGF plasma level increased strikingly in some ALS patients undergoing exercise [240]. Interestingly, an increased expression of VEGF mRNA with exercise has been demonstrated in muscle of normal exercising mice [245].

A correct evaluation of the results from the above studies should consider some methodological issues, namely: limited number of ALS patients and controls included; low sensitivity of the ELISA kit used; possible influence on the results from the cycles of freezing and thawing of biological samples [246–248].

An immunohistochemical study reported that VEGFR-2 immunostaining in neuropil was decreased in the spinal cord of ALS patients when compared to controls [210, 249]. These results reinforce the hypothesis that the reduction of the VEGF signaling may play a role in the pathogenesis of ALS [246].

## 5. Therapeutical Potential of VEGF in ALS

There is a great potential of VEGF treatment in ALS; Table 5 summarizes its potential routes for administration and delivery.

### 5.1. VEGF Gene and VEGF Protein Delivery in Central Nervous System (CNS)

Several studies used rodent ALS models for therapeutic purposes. After intracerebroventricular administration of recombinant VEGF in mice with ALS, it was observed delayed disease onset and prolonged survival [211], but systemic administration was noneffective. It was demonstrated that VEGF injected in the intracerebroventricular space is able to be transported anterogradely to the brainstem and lumbar spinal neurons, hence preserving the neuromuscular junctions and improving functional outcome [211, 250]. The intracerebroventricular administration of VEGF also induces the expression of GluR2 in the ventral spinal cord of rats, which demonstrates the protective effect of VEGF on motor neurons [251]. It was also shown that astrocytes are capable of protecting against excitotoxicity by inducing the expression of GluR2 on motor neurons, while this feature is not present in mSOD1 mice [252]. Motor neurons express lower levels of GluR2 subunit leaving them more vulnerable to AMPA receptor-mediated excitotoxicity [251, 253]. Administration of VEGF by intraperitoneal injections in SOD1^G93A^ transgenic mouse showed muscle weakness delay and prolonged survival in treated mice [254]. Intranasal administration of VEGF may provide an effective alternative. In one study, it was reported that after intranasal administration of VEGF in adult Sprague-Dawley rats, the highest tissue delivery was found in the trigeminal nerve, followed by the optic nerve, olfactory bulbs, olfactory tubercle, striatum, medulla, frontal cortex, midbrain, thalamus, hippocampus, and cerebellum [255].

Positive studies with VEGF were found in other studies, including one from Azzouz et al., which used a lentiviral vector pseudotyped with the rabies G envelope protein (EIAV vectors). Intramuscular administration of the vector permitted its retrograde transportation to the motor neuron, resulting in slower disease progression of the SOD1^G93A^ mice model, even when treatment was started at the onset of paralysis [41]. A more recent study with SOD1^G93A^ mice injected with adeno-associated virus serotype 4 VEGF vector showed promising results. Intracerebroventricular injection of AAV4-VEGF-165 led to significant extension of survival [198, 256]. By adenoviral vector it is possible to get an upregulation of VEGF expression indirectly and remotely delivered, through the use of a zinc finger protein. A study used engineered zinc-finger protein into a vector Ad-32-Flag-Ep65 (AD-p65), allowing to upregulate the expression of endogenous VEGF (VEGF-ZFP) in a laryngeal nerve (RLN)-crush injury model. The mice injected with adenovirus engineered ZFP-VEGF presented improvement in nerve regeneration, which led to a more rapid recovery when compared to controls [257]. Kliem and colleagues used a plasmid encoding zinc finger transcription factor protein (ZFP-TF) engineered to induce VEGF expression in mSOD1 rat model. The plasmid was administered intramuscularly in the lateral and medial gastrocnemius muscles at 80 days of age (prior to initiation of motor neuron degeneration) and weekly for a period of 6 weeks. Unilateral intramuscular delivery of VEGF in engineered zinc finger transcription factor preserved ipsilateral hindlimb grip strength and rotarod improved performance when compared to controls. Once the therapy was directed only to a single muscle, the investigators observed no change relative to the weight and the onset of disease of the mice [258].

Using a mouse NSC34 motor neuron-like cell culture system, following administration of an adenovirus vector in cultures containing mutant SOD1^G93A^, VEGF increased neuronal survival. However, the pretreated cells with VEGF displayed a dose-dependent resistance to oxidative damage, with the activation of both MAPK and PI3K pathways. The protective effects were mediated via VEGF PI3K activity [215].

The role of receptors as therapeutic targets requires further investigation. In an experimental study, antisense oligodeoxynucleotides against the VEGFR-2 were administrated in mice subjected to hypoxia, which resulted in the loss of nearly 50% of the motor neurons. This study demonstrates the direct neuroprotective activity of VEGFR-2. Motor neurons are more susceptible to degeneration when inhibiting the activation of AKT and ERK pathways, under conditions of hypoxia [259].

### 5.2. Role of VEGF: Use of Stem Cell Therapy in ALS

#### 5.2.1. Animal and Human Neural Stem Cells (NSCs)

Neural stem cells (NSCs) have an intrinsic ability to “rescue” dysfunctional neurons and to stimulate preservation, remodeling, and/or regeneration of host-derived neural circuitry [260]. In a meta-analysis, Teng and colleagues evaluated the effects of therapy of mouse and human NSCs transplantation in mSOD1 mice. Only one injection resulted in an extensive and robust integration of donor cells in the lumbar cord. NSCs promoted an increase in the secretion of trophic factors that contributed to the survival motor neuron, by reducing inflammation and astrogliosis, related to trophic factors production. It was observed, in the mice model, improved motor performance and respiratory function, slower disease progression, and delayed-onset disease [260, 261].

After intrathecal administration of the NCSs engineered to overexpressing human VEGF gene (HB1.F3.VEGF) in SOD1^G93A^ mouse model, there was a delay in disease onset and the survival was prolonged without significant adverse effects when compared to control animals. Transplanted cells were found in the anterior horn and differentiated into motor neurons. The neuroprotective effect was achieved by the production of antiapoptotic proteins (Bcl-2and Bcl-XL) and molecules promoting cell survival and downregulating proapoptotic proteins (Caspase-3 and BAX) [262].

#### 5.2.2. Human Mesenchymal Stem Cells (hMSCs)

A study with single human mesenchymal stem cells (hMSCs) showed that transplantation of hMSCs had no beneficial effect in mSOD1 mice. However, multiple transplantations allowed decreased motor neuron loss, enhanced motor performance, and increased survival in mSOD1 mice, although only a restricted number of cells migrated into the lumbar spinal cord parenchyma [263].

In 2013, Krakora et al. used hMSCs expressing both GDNF (hMSC-GDNF) and VEGF (hMSC-VEGF) that were injected into SOD1^G93A^ rats, which slowed motor function loss, protected neuromuscular junctions (NMJs), and prolonged lifespan. A synergistic effect was found when using combined delivery of these factors, compared to the single administration of each of these neurotrophic factors [264].

#### 5.2.3. Human Adipose-Derived MSCs Stem Cells

It was demonstrated that the soluble growth factors (VEGF, HGF, and IGF-1) released by human adipose-derived stem cells (hADSCs) on primary astrocytes cultured from SOD1^G93A^ mice significantly upregulate the expression of astrocytes GLT1 [265]. Marconi et al. demonstrated that the systemic injection of ADSCs in mSOD1 mice delayed disease onset and slowed deterioration of motor performance. This and other studies reinforce the idea that ADSCs can produce a number of neurotrophic factors (VEGF and IGF-1) capable of supporting neuronal survival [266–268].

#### 5.2.4. Bone Marrow Stem Cells

Corti and colleagues evaluated the effect of cell therapy using intravascular injection of C-kit (+) stem/progenitor cells in the spinal cord of SOD1^G93A^ mice. The transplanted stem cells caused a delay in disease progression, prolonged lifespan, promoted survival of motor neurons, and improved neuromuscular function. Neuroprotection resulted partially from the preservation of glutamate transporter GLT-1 levels, reduction of microgliosis, and increase of VEGF and angiopoietin 2 [269, 270].

A study was performed using genetically modified blood mononuclear cells from human umbilical cord blood (HUCB). They were transiently transfected by electroporation with VEGF-A in SOD1^G93A^ mice. These cells were retroorbitally administered via injection in presymptomatic phase (22–25 weeks). The results showed a differentiation of transplanted cells HUCB in vascular ECs and an increased survival of motor neuron in these mice [271].

## 6. Use of VEGF in ALS Patients

Trials testing VEGF for the treatment of neurodegenerative diseases, including ALS, have been considered after some positive experimental studies in animal models. Regarding human studies, it is crucial to evaluate safety. Particular care should be taken regarding complex VEGF effects on angiogenesis, inflammation, and capillary permeability [167, 221, 272, 273]. A critical problem is to define a treatment window of VEGF in humans, taking into account that in SOD1^G93A^ mice ICV administration at a dosage of 0.2 *μ*g VEGF·kg^−1^·day^−1^ a day conferred neuroprotection, without undesirable inflammatory or angiogenic effects [90, 211]. Another therapeutic pathway to explore is VEGF-B, a factor with few angiogenic potential. Some studies have reported that it has a neuroprotective effect with higher potential security when compared to VEGF-A [220].

Another issue that deserves to be addressed is the emerging need for standardization of analytical procedures in various biological samples, when it becomes necessary to determine VEGF levels. Further technological advances for determining other VEGF isoforms and their receptors (namely VEGF-B) are necessary, with greater sensitivity than the conventional ELISA.

The company NeuroNova AB has been sponsoring clinical tests to develop a therapy based on direct intracerebroventricular administration of VEGF_165_ in ALS patients (by means of an FDA-approved and CE-marked pump and a specialized catheter for protein drug delivery directly into the brain by ICV infusion, sNN0029). Following the phase-1 trial (NCT00800501, a double-blind, randomised, parallel group safety and tolerability study), there is an open label safety and tolerability continuation study to further evaluate safety and tolerability, as well as motor function and survival in patients who participated in the previous study (NCT01384162).

A phase-2 trial in ALS patients is sponsored by Sangamo BioSciences (NCT00748501)—*Clinical Trial of SB-509 in Subjects with Amyotrophic Lateral Sclerosis (ALS).* Sangamo's drug, SB-509, is an injectable formulation of a plasmid encoding a zinc finger DNA-binding protein transcription factor (ZFP TF(TM)-Sangamo BioSciences) designed to upregulate the expression of the gene encoding VEGF-A. It was designed as a randomized repeat-dosing, open-label, multicenter trial to evaluate the effect of intramuscular administration of SB-509 on the progression of the disease, as measured by the ALS Functional Rating Scale-Revised (ALSFRS-R). ALSFRS-R is a validated rating instrument for monitoring the progression of disability in patients with ALS. In addition to gathering data on safety and tolerability of SB-509 in ALS patients, data will be collected to evaluate the effect of SB-509 on additional clinical measures, forced vital capacity, neurophysiologic index [274], manual muscle test, and survival. The study is completed but no results have been released.

A randomized, blinded trial of intramuscular gene transfer using plasmid VEGF to treat diabetic polyneuropathy was done [275]. Thirty-nine diabetic patients with polyneuropathy were randomized to receive a VEGF-to-placebo ratio of 3 : 1. Three sets of injections were given at eight standardized sites adjacent to the sciatic, peroneal, and tibial nerves of one leg. Primary outcomes were changes in symptom score at 6 months and a prespecified overall clinical and electrophysiological improvement score. Intramuscular plasmid VEGF gene transfer tended to improve primary outcome, without major safety issues. The possibility to translate this approach to ALS patients is an open issue.

Further studies should be considered to test the use of stem cells combined with neurotrophic factors, as it seems to be a potential therapy for ALS. Those studies should be carried out under strict monitoring, thereby ensuring that the therapy is safe in humans.

Although VEGF is a very promising compound, there is a major challenge ahead that is to translate the basic science breakthroughs into clinical practice.

## 7. Concluding Remarks

No effective treatment able to stop disease progression is available for ALS. A large number of conventional trials have been negative, involving drugs with quite different properties. More challenging steps are necessary, testing breakthrough compounds and using more advanced methods for drug delivery.

The history of VEGF in medicine is not long but is rich and complex. The emerging evidence regarding the roles of this neurotrophic factor provides a strong rational for testing VEGF in neurodegenerative disorders. The future has started already and preliminary tests on the potential therapeutic efficacy of VEGF have been tested in patients with ALS. The future will show the right track, or at least the wrong ones. Anyway, it is relevant for the ALS community to know more on VEGF.

## Figures and Tables

**Table 1:** Different members of ligands of VEGF family and characteristics and properties.

Ligand	Chromosomal localization	Protein molecular weight	Isoforms	Expression sites High levels of expression	Role/remarks	Receptors	References
VEGF-A	**6p21.3** (8 exons, 7 introns)	Dimeric glycoprotein 33–42 kDa Precursor protein-232 aa	Human VEGF_121_ (VEGF_121b_*) VEGF_145_ (VEGF_145b_*) VEGF_148_, VEGF_165_ (VEGF_165b_*) VEGF_183_ (VEGF_183b_*) VEGF_189_ (VEGF_189b_*) VEGF_206_ **Mice**: VEGF_120_, VEGF_164_, VEGF_188_ (alternative mRNA splicing)	Adrenal gland, lung kidney, heart	Angiogenesis, survival, neuronal development, increased migration, and proliferation of endothelial cells Vasodilation (by inducing the endothelial nitric oxide synthase and increasing nitric oxide production) Monocyte motility	VEGFR-1 sVEGFR-1 VEGFR-2 NRP-1 NRP-2 (VEGF_145_, VEGF_165_)	[46, 49, 50, 58, 63, 70]

Placental growth factor (PlGF)	14q24 (7 exons)	Homodimeric glycoprotein 45 kDa 131 aa (PlGF-1) 152 aa (PlGF-2)	PLGF-1 (PLGF 131) PLGF-2 (PLGF 152) PLGF-3 (PLGF 203) PLGF-4 (PLGF 224) (alternative mRNA splicing)	Placenta, lungs, heart, skeletal muscle, thyroid gland, adipose tissue, retina, skin, ECs, trophoblasts monocytes, erythroid cells	Regulation of vascular differentiation, vasculogenesis, angiogenesis during inflammation, wound healing, ischemia, and cancer	VEGFR-1 NRP-1 (PLGF-2)	[49, 56, 57, 59–65, 70]

VEGF-B	11q13 (7 exons, 6 introns)	21 kDa; 167 aa (VEGF-B_167_) 32 kDa; 186 aa (VEGF-B_186_) 188 aa	VEGF-B_167_ and VEGF-B_186_ (alternative mRNA splicing)	Myocardium, skeletal muscle, pancreas, neural tissues (retina, brain, and spinal cord), smooth muscle cells of large vessels, prostate, brown fat	Embryonic angiogenesis, mitogenic factor for human ECs, strong cell survival factor for different types of cells: neurons, vascular cells (pericytes, EC, and smooth muscle cells), and myocytes	VEGFR-1 sVEGFR-1 NRP-1	[50, 56, 57, 63, 66–74, 216]

VEGF-C	4q34 (7 exons)	29–31 kDa 20 kDa mature protein 399 aa	VEGF-C_62_, VEGF-C_129_, and VEGF-C_184_ (from immortalized mouse)-splicing isoforms Proteolytic mechanisms	**High levels**: heart, ovary, placenta, skeletal muscle, thyroid gland, and small intestine **Modest transcripts**: kidney, lung, pancreas, prostate, spleen	Angiogenesis and lymphangiogenesis (mitogenesis, the mature form of VEGF-C induces migration and survival of ECs and induces selective lymphangiogenesis without accompanying angiogenesis)	VEGFR-2 VEGFR-3	[50, 56, 57, 63, 70, 72, 76, 77, 79–81, 276]

VEGF-D	Xp22.31 (7 exons)	Glycoprotein human cDNA encodes protein 354 aa	Two isoforms, Proteolytic mechanisms	**High levels**: Heart, lungs, skeletal muscle, colon, and small intestine **Modest transcripts**: ovary, pancreas, prostate, spleen, testes	Angiogenesis and lymphangiogenesis (induces proliferation, survival, and migration of lymphatic ECs)	VEGFR-2 VEGFR-3	[50, 56, 57, 63, 70, 72, 75–82, 85]

VEGF-E	Non-human Orf parapoxvirus	120–140 aa	VEGF-E_NZ-2_ VEGF-E_NZ-7_ VEGF-E_NZ-10_ VEGF-E_D1701 _ VEGF-E_VR634_	ds DNA parapoxvirus	Potent angiogenic activity growth, vascular permeability, proliferation, migration, sprouting, and mitotic activity of vascular ECs, stimulates chemotaxis	VEGFR-2 NRP-1	[49, 56, 57, 63, 81, 86, 92]

VEGF-F/Trimeresurus flavoviridis svVEGF (T.f. svVEGF)	Non-human	110–122 aa Functions as dimmers-2 proteins: Vammin (110 residues aa) VR-1 (109 residues aa)		Isolated from snake venom: svVEGF from Bothrops insularis TfsvVEGF (Trimeresurus flavoviridis svVEGF) from pit vipers	Weak angiogenic activity Strong vascular permeability activity Induces hypotension	VEGFR-1	[49, 56, 63, 81, 87]

aa: amino acids.

Ec: endothelial cell.

VEGF_*XXX*_: the number of amino acids on the polypeptide chain.

*VEGF_XXXb_: Antiangiogenic isoforms.

**Table 2:** Different members of receptors of VEGF family and some characteristics and properties.

	Receptor	Chromosomal localization	Molecular weight	Properties	High levels of expression	Role	Binding Protein	References
VEGF receptors tyrosine kinases	VEGFR-1 (fms-like tyrosine kinase-1 or Flt1)	13q12-q13 (30 exons)	Glycoprotein 180 kDa 1338 aa	Extracellular domain-7 immunoglobulin-like domains (alternative mRNA splicing encodes a soluble truncated form-sVEGFR-1)	Endothelial cells, pericytes, placental trophoblasts osteoblasts, renal mesangial cells, monocytes/macrophages, some hematopoietic stem cells	Regulating angiogenesis (plays a negative role in angiogenesis-suppressing proangiogenic signals in the embryo). Expression is upregulated during angiogenesis and hypoxia. Activation of macrophages	VEGF-A VEGF-B PLGF-1,2	[52, 56, 57, 63, 70, 92–98]
	VEGFR-2 (KDR in humans, Flk1 in mice	4q11-q12	Glycoprotein 200–300 kDa 1356 aa	Extracellular domain-7 immunoglobulin-like domains (alternative mRNA splicing encodes a soluble truncated form-sVEGFR-2)	Osteoblasts, cells of the pancreatic duct, neuronal cells, retinal progenitor cells, hematopoietic stem cells, megakaryocytes, nervous system	Regulating angiogenesis, mitogenesis, migration, and permeability of the ECs. PAF production by ECs (stimulating mitosis, survival, migration, and increasing vascular permeability)	VEGF-A (lower affinity) VEGF-C VEGF-D VEGF-E	[50, 52, 56, 57, 63, 70, 85, 89–92, 99–106]
	VEGFR-3 (fms-like-tyrosine kinase (Flt)-4)	5q33-qter	195 kDa	Extracellular domain-6 immunoglobulin-like domains Two human isoforms (alternative mRNA splicing and differing in their C-terminal)	Osteoblasts, neuronal progenitors cells, macrophages	Lymphangiogenesis, mitogenesis, differentiation, and survival of lymphatic endothelial cells. Hematopoiesis and vasculoangiogenesis. Role in development of the cardiovascular system. Angiogenesis (early embryo).	VEGF-C VEGF-D	[50, 52, 55, 57, 63, 70, 81, 85, 90, 91, 107, 109–115]
VEGF receptors nontyrosine kinases	Neuropilin NRP-1	10p12 (contains 17 exons)	Glycoprotein 130–140 kDa	Semaphorin family VEGF family	Axons (expressed in the tips of growing axons), certain classes of neurons, ECs of blood vessels, heart, placenta, lungs, liver, kidney, skeletal and pancreas, bone marrow fibroblasts, adipocytes, dendritic immune cells, osteoblasts, renal mesangial, renal glomerular epithelial cells expressed in arteries	Development of neuronal guidance. Angiogenesis and immunology involved in cardiovascular system. Neuropilin potentiate VEGF signaling (acting as co-receptors for VEGF receptors). Axon growth and guidance in the developing embryo (chemorepulsive axon guidance molecules capable of collapsing axonal growth cones and repelling axons of ganglia during neurogenesis)	VEGF_165_ VEGF-B VEGF-E PLGF-2 Semaphorin-3A Semaphorin-3C Semaphorin-3F Pro-VEGF-C Pro-VEGF-D	[46, 53, 56, 57, 63, 70, 90, 116–126, 128]
	Neuropilin NRP-2	2q34 (contains17 exons)	Glycoprotein 130–140 kDa	Semaphorin family VEGF family	Expressed in venous and lymphatic vessels Neuronal cells	Development of neuronal guidance, angiogenesis, and immunology, involved in cardiovascular system Neuropilin potentiate VEGF signaling (acting as co-receptors for VEGF receptors) chemorepulsive axon guidance molecules (capable of collapsing axonal growth cones and repelling axons of ganglia during neurogenesis)	VEGF_165_ VEGF_145_ Pro- and mature VEGF-C Pro-VEGF-D Semaphorin-3C Semaphorin-3F	[46, 56, 57, 63, 70, 90, 116–124, 128]

PAF: platelet activating factor.

**Table 3:** VEGF neurotrophic activity (adapted from Keifer et al., 2014 [198]).

VEGF factor	Role	References
VEGF-A	Neurogenesis	[103, 277, 278]
	Nerve migration	[279]
	Axonal guidance	[167, 280, 281]
	Survival/neuroprotection	[39, 41, 282]
	Protective effects in adverse conditions: Hypoxia Serum deprivation Excitotoxicity Mechanical trauma Chemical toxicity	[36, 37, 182, 183, 212, 251, 283, 284]

VEGF-B	Survival: retinal and cortical neurons	[165, 217]

VEGF-C	Nervous system development Proliferation and activation of astroglia and microglia	[113, 285, 286]

VEGF-D	Survival/neuroprotection Development of embryonic stem cells	[279, 287]

PLGF	Survival/neuroprotection	[288, 289]

**Table 4:** VEGF activity in dendritogenesis, synaptic plasticity, and axonal growth in adult brain (adapted from Mackenzie and Ruhrberg, 2012 [167]).

VEGF	Role	References
VEGF	Dendritogenesis	[167, 172, 282, 290–292]
	Synaptic plasticity	[90, 172, 290–295]
	Axonal growth	[102, 167, 172, 280–282, 296–298]

**Table 5:** Summary of themode of administration and delivery form ofVEGF, its advantages, and disadvantages (adapted from Keifer et al., 2014 [198]).

Administration	
Direct	Indirect/Remote
(i) Direct access to the target area (ii) Decreases complications in areas outside the therapeutic scope (iii) Smaller dose required	(i) The product is delivered in the CNS secondarily (ii) Necessary higher dosages (iii) Less invasive administration (iv) Product should have the ability to cross the BBB, pial barrier, and be transported retrogradely by CNS

Delivery	

Cells that produce or secrete VEGF-A Use of a viral vector or nonviral to produce VEGF-A or a protein that enhances expression of endogenous VEGF-A (transcription factor) Immediate delivery of VEGF-A protein to the target region	
